# Modification of *Saccharomyces cerevisiae* Cells with Metal Hexacyanoferrates for the Construction of a Yeast-Based Fuel Cell

**DOI:** 10.3390/molecules30010137

**Published:** 2025-01-01

**Authors:** Gabija Adomaitė, Povilas Virbickas, Aušra Valiūnienė

**Affiliations:** 1 Institute of Chemistry, Faculty of Chemistry and Geosciences, Vilnius University, Naugarduko Str. 24, LT-03225 Vilnius, Lithuania; gabija.kavaliauskaite@chgf.stud.vu.lt (G.A.); povilas.virbickas@chgf.vu.lt (P.V.); 2 Department of Biomedical Science, Faculty of Health and Society, Malmö University, SE-205 06 Malmö, Sweden; 3 Biofilms—Research Center for Biointerfaces, Malmö University, SE-205 06 Malmö, Sweden; 4 State Research Institute Center for Physical Sciences and Technology, Sauletekio Ave. 3, LT-10257 Vilnius, Lithuania

**Keywords:** Prussian blue, nickel hexacyanoferrate, biofuel cell, yeast cells, *Saccharomyces cerevisiae*

## Abstract

This research presents a simple procedure for chemically modifying yeast (*Saccharomyces cerevisiae*) cells with nickel hexacyanoferrate (NiHCF) and ferric hexacyanoferrate, also known as Prussian blue (PB), to increase the conductivity of the yeast cell wall. Using linear sweep voltammetry, NiHCF-modified yeast and PB-modified yeast (NiHCF/yeast and PB/yeast, respectively) were found to have better cell wall conductivity in [Fe(CN)_6_]^3−^ and glucose-containing phosphate-buffered solution than unmodified yeast. Spectrophotometric analysis showed that the modification of yeast cells with NiHCF had a less harmful effect on yeast cell viability than the modification of yeast cells with PB. The use of NiHCF/yeast and PB/yeast cells in the construction of a yeast-based fuel cell allowed the maximum power densities of 62.66 mW/m^2^ and 94.09 mW/m^2^ to be achieved. These values were much higher than those obtained using unmodified yeast cells (42.25 mW/m^2^). NiHCF/yeast and PB/yeast fuel cells were renewed by replenishing the yeast suspension in the anolyte or the FeCl_3_ salt in the catholyte. This allowed 77.4% and 50.1% of the initial maximum power density of the fuel cells to be achieved.

## 1. Introduction

Biofuel cells (BFCs) are one of the potential alternatives to conventional power generation [1,2,3,4]. Yeast-based fuel cells are particularly attractive due to their simplicity of design and cost-effectiveness, which is due to the availability and affordability of baker’s yeast (*Saccharomyces cerevisiae*) [3]. Yeast cells are one of the most scientifically studied cells [5]; thus, baker’s yeast containing bioelectronic systems is common. However, the limited efficiency of charge transfer through the yeast wall, resulting in a restricted transfer of metabolically generated electrons from yeast to an anode, is a crucial parameter [6] that needs to be improved [7,8]. Methods to improve the conductivity of *Saccharomyces cerevisiae* cells have been proposed over time [9,10]. However, there is a great need for a simple, practical, and non-demanding method of yeast modification to improve the electrical output of yeast-based fuel cells [6].

Research on electrochemical catalytic processes and the use of inorganic catalysts, including the operation of biofuel cells, is gaining great scientific interest [11,12,13]. Recently, a yeast modification method involving the incorporation of a well-known inorganic electrocatalyst, Prussian blue (PB) [14], into the internal structure of yeast cells has been investigated [1], and the results have shown that the modification of yeast cells with PB increases the permeability of the yeast cell wall and consequently results in higher power densities of a BFC compared to a BFC of the same design using unmodified yeast cells [1,15,16,17] Unfortunately, the biofuel cell based on yeast cells modified with PB did not exhibit the expected longevity [1], which may be related to the reduction in yeast cell viability due to the cytotoxic effect of Fe^3+^ ion (and, possibly, [Fe(CN)_6_]^3−^) used during the PB modification of yeast cells [18,19]. Therefore, it may be worthwhile to consider whether the modification of yeast cells with other Fe^3+^-free PB analogs, also known as transition metal hexacyanoferrates (MeHCFs), would improve the performance of the yeast-based fuel cells, and how this would affect the viability of the yeast cells and the longevity of yeast-based fuel cells.

In order to reduce the negative effect of yeast cell modification with MeHCFs on the viability of the yeast cell, the toxic effect of MeHCF-forming metal ions should first be considered. In contrast to some other MeHCF-forming transition metal ions (e.g., Cu^2+^, Pb^2+^, and Ag^+^), Ni ions, at least in the divalent state, do not seem to affect the viability of the yeast *Saccharomyces cerevisiae* at concentrations below 200 mM [20,21], and, to the best of our knowledge, the effect of Ni^3+^ on yeast viability has not yet been determined. Therefore, experiments with nickel hexacyanoferrate (NiHCF) may be a promising way to develop efficient yeast-based fuel cells while avoiding (or reducing) the negative effect of chemical modification on yeast cell viability. Moreover, the application of NiHCF may improve the properties of BFCs based on yeast cells modified with PB [1], as NiHCF is known to have a property of low voltage hysteresis [22]. Voltage hysteresis is a material property where the application or removal of voltage to systems occurs after a delay and is highly avoidable in battery design [23,24,25].

This research study presents a novel, low-cost, and simple method of modifying *Saccharomyces cerevisiae* cells with PB and NiHCF with the objective of enhancing the electric output of yeast-based fuel cells. The presented modification method can be regarded as a cost-effective alternative for yeast cell modification, as the utilized materials are readily commercially available and do not necessitate additional modifications in comparison to the polymers and nanomaterials employed in other studies [7,10]. Considering that the chemical modification procedure may have a negative impact on the PB/yeast or NiHCF/yeast viability, the modified yeast cells were studied using a spectrophotometric technique proposed by A. Matsumoto et al. [26]. Linear sweep voltammetry was used to investigate the ability of unmodified, PB-modified, and NiHCF-modified yeast to reduce the charge transfer mediator [Fe(CN)_6_]^3−^ [27], as the efficient transfer of metabolically generated electrons from yeast to the charge transfer mediator is crucial for the effective operation of yeast-based fuel cells. It has been found that the electrochemical properties of the proposed yeast-based fuel cells are equivalent to those of yeast-based fuel cells that are more complex in their construction [2,7,9,10]. Additionally, two types of yeast-based fuel cell renewal are presented in this paper to ensure that the proposed yeast-based fuel cell is considered an environmentally viable option.

## 2. Results

### 2.1. Effect of Yeast Modification with Metal Hexacyanoferrates on Yeast Viability

It has been established [7] that one of the major challenges in improving the performance of yeast-based fuel cells is the limited permeability of the yeast cell wall to charge transfer mediators (e.g., ferricyanide) and its low electrical conductivity. Limited cell wall permeability limits the ability of charge transfer mediators to diffuse through the cell wall and exchange electrons with oxidoreductases located in the periplasmic space of yeast cells, while limited electrical conductivity prevents charge transfer from oxidoreductases to charge transfer mediators located outside the cell. These limitations on charge transfer from the oxidoreductases to the charge transfer mediator are expected to reduce the power generated by a yeast-based fuel cell. However, charge transfer can be facilitated by the chemical modification of the yeast cell wall, resulting in improved permeability and/or electrical conductivity. Therefore, to improve the performance of a yeast-based fuel cell, the yeast wall can be modified with transition metal hexacyanoferrates [1], polypyrrole, polydopamine [28], or carbon nanotubes [9]. However, the chemical modification of the yeast wall can negatively affect the viability of yeast cells [6], which could lead to worse efficiency of the biofuel cell. Therefore, the successful chemical modification of yeast cells should result in (i) a significant increase in the efficiency of electron transfer from yeast cell enzymes to the charge transfer mediator and (ii) maximally preserved yeast cell viability.

In this work, the viability of NiHCF/yeast and PB/yeast was investigated using the spectrophotometric technique proposed by A. Matsumoto et al. [26]. This spectrophotometric technique evaluates the viability of yeast cells based on the absorption of the supernatant, i.e., non-viable yeast cells absorb more methylene blue (MB) than living cells, resulting in less optical absorption of the supernatant. Thus, the absorption of the supernatant obtained by the centrifugation of freshly cultured yeast cells should be higher than that of the supernatant obtained by the centrifugation of non-viable yeast cells. The percent of viable yeast cells in a sample can be calculated according to Equation (1) [26]:

(1) $$\mathrm{Viable}\;\mathrm{yeast}\;\mathrm{cells},\;\%\;=\frac{ABS_{s}-ABS_{non-viable}}{ABS_{viable}-ABS_{non-viable}}$$

where *ABS_s_* is the maximum absorption value of the supernatant peak obtained by centrifugation of freshly cultivated NiHCH/yeast or PB/yeast; *ABS_non-viable_* is the absorption of supernatant obtained by the centrifugation of non-viable NiHCF/yeast or non-viable PB/yeast; and *ABS_viable_* is the absorption value of supernatant obtained by the centrifugation of freshly cultivated unmodified yeast.

**Figure 1:**
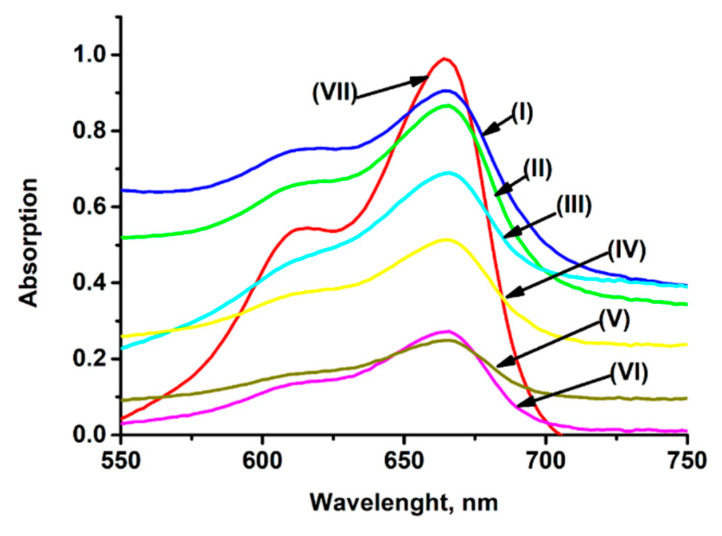
Absorption spectra of the supernatant obtained by centrifugation of (I) freshly cultivated unmodified yeast cells; (II) freshly cultivated NiHCF/yeast cells; (III) freshly cultivated PB/yeast cells; (IV) non-viable unmodified yeast cells; (V) non-viable NiHCF/yeast cells; (VI) non-viable PB/yeast cells; “base solution” containing MB and trisodium citrate, without yeast cells (VII).

Figure 1 shows the absorption spectra of the supernatant obtained by the centrifugation of freshly cultivated unmodified yeast cells (curve I), freshly cultivated NiHCF/yeast cells (curve II), freshly cultivated PB/yeast cells (curve III), non-viable unmodified yeast cells (curve IV), non-viable NiHCF/yeast cells (curve V), and non-viable PB/yeast cells (curve VI). The control measurement of the absorption of the “base solution” (containing MB and trisodium citrate, without yeast cells) is also shown in Figure 1 (curve VII).

The absorption of the supernatant after the interaction of differently modified yeast cells was reduced due to the amount of non-viable yeast cells in the sample (Figure 1). According to Equation (1), the viability of NiHCF/yeast was approximately 100%, whereas the viability of PB/yeast was 61%. This result indicates that the NiHCF/yeast and the PB/yeast cells can ensure the successful operation of a yeast-based fuel cell.

### 2.2. Effect of Yeast Modification with Metal Hexacyanoferrates on Electron Transfer Capability

Additionally, the effect of the chemical modification of yeast cells on the efficiency of electron transfer through the yeast cell wall also needs to be determined. For this reason, a linear sweep voltammetry study was carried out on PBS-based suspensions containing glucose, yeast cells (NiHCF-modified, PB-modified, or unmodified), and the charge transfer mediator [Fe(CN)_6_]^3−^. The same experiments were carried out in a control solution (without yeast cells) containing PBS, glucose, and [Fe(CN)_6_]^3−^. According to Equation (2) [27], the metabolically generated electrons from the yeast cells can be transferred to the charge transfer mediator [Fe(CN)_6_]^3−^ to form [Fe(CN)_6_]^4−^ ions, which can then be oxidized at a Pt electrode (Equation (3)).

[Fe(CN)_6_]^3−^ + e^−^ (from yeast cells) → [Fe(CN)_6_]^4−^ (2)

[Fe(CN)_6_]^4−^ → [Fe(CN)_6_]^3−^ + e^−^ (3)

**Figure 2:**
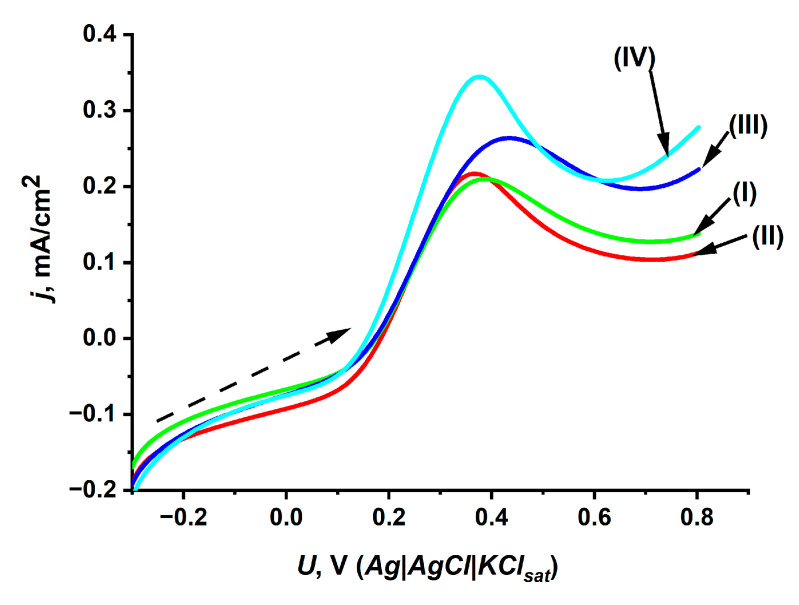
Linear sweep voltammograms of Pt electrode determined in PBS-based solution of K_3_[Fe(CN)_6_] (5 mM) containing (I) glucose (0.1 M), (II) unmodified yeast (1 g/mL) and glucose (0.1 M), (III) NiHCF/yeast and glucose (0.1 M), and (IV) PB/yeast and glucose (0.1 M). The potential scan rate was 50 mV/s.

Electrochemical measurements (Figure 2) show that the oxidation of [Fe(CN)_6_]^4−^ in suspensions containing NiHCF/yeast (Figure 2, curve III) and PB/yeast (Figure 2, curve IV) resulted in anodic peak currents of 528 μA/cm^2^ and 689 μA/cm^2^, respectively. The lower peak current of 420 μA/cm^2^ was obtained in the PBS-based solution containing unmodified yeast (Figure 2, curve II). The higher anodic peak current values in suspensions containing NiHCF/yeast and PB/yeast cells can be related to NiHCF and PB acting as charge transfer mediators [1]—the hexacyanoferrate group of PB and NiHCF could be involved in the transport of metabolically generated electrons from enzymes located in the periplasm of yeast cells to the [Fe(CN)_6_]^3−^ ions dissolved in the solution. The enhanced transport of electrons from the yeast cells to [Fe(CN)_6_]^3−^ is expected to increase the concentration of [Fe(CN)_6_]^4−^, resulting in a higher value of anodic peak current (Figure 2, curves III and IV).

### 2.3. Construction and Investigation of the Biofuel Cells

The increased anodic peak currents in the solutions containing modified yeast can be considered a promising characteristic for the construction of biofuel cells (BFCs). Therefore, the NiHCF/yeast and PB/yeast were used to design the BFCs in this study.

**Figure 3:**
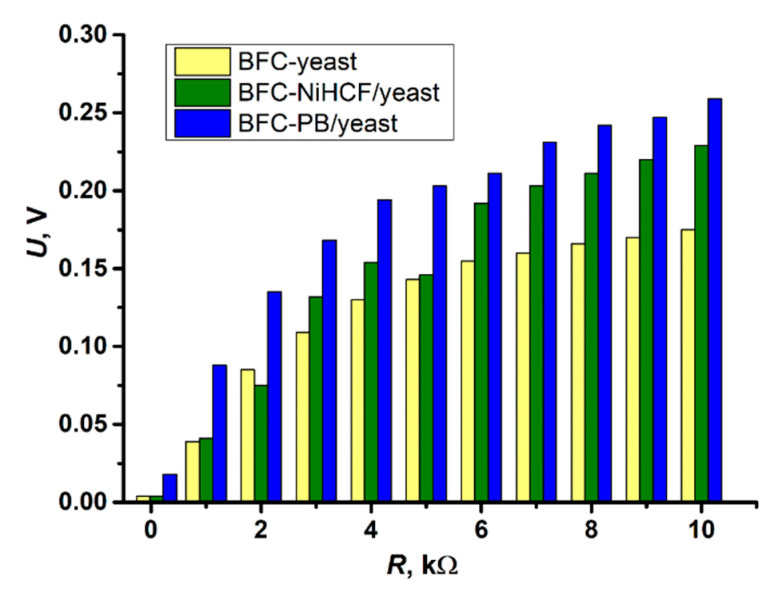
Change in voltage across the applied resistance measured for biofuel cells based on unmodified yeast (BFC yeast), NiHCF/yeast (BFC NiHCF/yeast), and PB/yeast (BFC PB/yeast) in the resistance series interval from 1 kΩ to 10 kΩ.

To obtain the voltages of the fuel cells, a series of resistors (ranging from 10 Ω to 94 MΩ) were inserted between the cathodic and anodic sides of BFCs based on unmodified yeast (BFC yeast), NiHCF/yeast (BFC NiHCF/yeast) and PB/yeast (BFC PB/yeast). Depending on the type of yeast cells used to construct the BFC, the change in resistance applied between the electrodes resulted in different voltages being produced (Figure 3). The BFC NiHCF/yeast resulted in a maximum voltage of 0.229 V at a 10 kΩ resistance load, which is 31% higher compared to the BFC yeast. In addition, the BFC PB/yeast resulted in a 48% higher maximum voltage of 0.259 V at a 10 kΩ resistance load compared to the BFC yeast. The overall voltages generated by the biofuel cells constructed in the current study are significantly higher than those obtained in our previous research [1], where unmodified and PB-modified yeast were used to construct a fuel cell. The higher voltages obtained in this study are probably related to the choice of Pt as the cathode and anode, in comparison with the previously used carbon electrodes [1], which are considered to be susceptible to spalling during the oxidation process and more prone to adsorb biochemical substances than chemically inert Pt electrodes [29].

**Figure 4:**
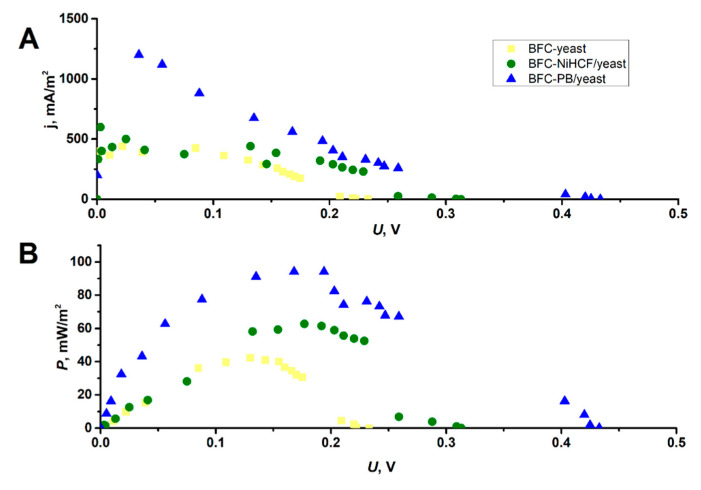
Investigation of the biofuel cells based on unmodified yeast (BFC yeast), NiHCF/yeast (BFC NiHCF/yeast), and PB/yeast (BFC PB/yeast): (**A**) dependency of the current densities (*j*, mA/m^2^) over the potential (*U*, V); (**B**) dependency of the power densities (*P*, mW/m^2^) over the potential (*U*, V).

The data from Figure 3 were further used to calculate the values of the current densities (*j*, mA/m^2^) and power densities (*P*, mW/m^2^) of the BFCs (Figure 4) using Equations (4) and (5), respectively.

*j* = (*U*·*A*)/*R* (4)

*P* = *U* · *j* (5)

where *j* is the current density (mA/m^2^), *U* is the potential difference between the cathode and the anode (V), *R* is the resistance (kΩ), *A* is the geometric surface area of the anode (1 × 10^−4^ m^2^), and *P* is the power density (mW/m^2^).

The maximum current density of BFC NiHCF/yeast was calculated (Equation (4)) to be 23% higher than that of BFC yeast, while the maximum current density of BFC PB/yeast was 65% higher than that of BFC yeast (Figure 4A). Accordingly, the maximum power density for BFC yeast was calculated (Equation (5)) to be lower (42.25 mW/m^2^), but BFC NiHCF/yeast and BFC PB/yeast reached the expected higher values of 62.66 mW/m^2^ and 94.09 mW/m^2^, respectively (Figure 4B). These values are in a similar power density range (60–155 mW/m^2^) to other BFCs described in the literature that were constructed similarly but not exactly as in this study [17,30,31,32]. The increased current and power densities observed in BFCs derived from modified yeast cells compared to BFC yeast can be attributed to the influence of MeHCF on the flow of electrons. As the MeHCFs are known to act as redox mediators [7], the presence of MeHCFs in a periplasmic area and cell wall of yeast is expected to facilitate the transfer of electrons from yeast oxidoreductases to the redox mediator ferricyanide, making the flow of electrons through the yeast cell wall less restricted. As a result, the accelerated electron flow leads to a higher current density and consequently a higher power density of BFCs.

### 2.4. Renewal of the Biofuel Cells

**Figure 5:**
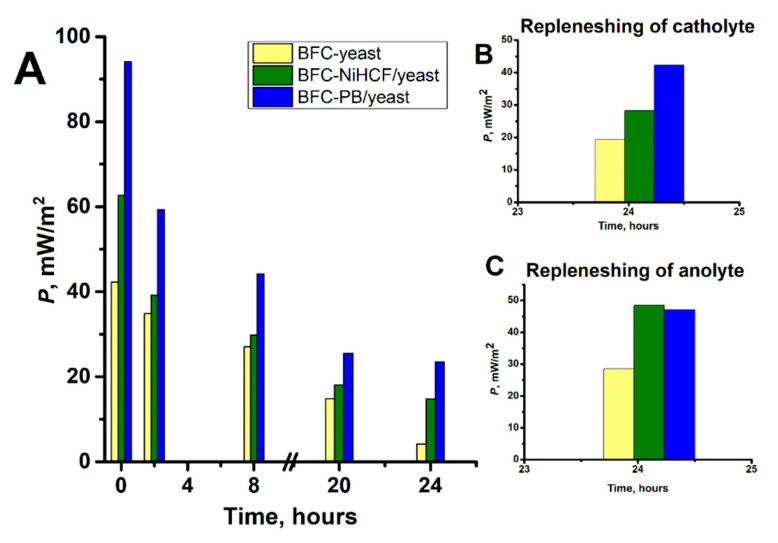
(**A**) Durability investigation of the biofuel cells visualized by plotting the power density (*P*) values vs. time; (**B**,**C**) values of *P* obtained after replenishing catholyte solution and anolyte suspension.

Further experiments were carried out to determine the change in power density of the BFC yeast, BFC NiHCF/yeast, and BFC PB/yeast over time (Figure 5A). It was determined that after 24 h of operation, the BFC yeast lost approximately 90% of its initial power density, while the BFC NiHCF/yeast and BFC PB/yeast lost approximately 75% of their initial power densities. It can be considered that as FeCl_3_ accepts the electrons produced by the metabolic reaction of the yeast cells and is reduced to FeCl_2_, the power density of the biofuel cells may start to decrease due to the reduced amount of Fe^3+^ ions near the cathode. With this in mind, we decided to renew all three types of biofuel cells (BFC yeast, BFC NiHCF/yeast, and BFC PB/yeast) after 24 h of operation by replenishing the used FeCl_3_ solution with freshly prepared, i.e., by renewing the catholyte. It was found that after the renewal of the catholyte, each biofuel cell returned to approximately 45% of its initial power density. To be more precise, after renewing the BFCs, the power densities were equal to 19.36 mW/m^2^ for the BFC yeast, 28.28 mW/m^2^ for the BFC NiHCF/yeast, and 42.25 mW/m^2^ for the BFC PB/yeast (Figure 5B). Overall, these results indicate that the BFCs can be renewed by replenishing the FeCl_3_ solution, but the results of this type of renewal are not entirely satisfactory. The limited efficiency of replenishing the FeCl_3_ solution is probably related to the changes in the catholyte suspension caused by the metabolic activity of the yeast, i.e., the reduced concentration of glucose and the presence of side products of yeast metabolism.

Another approach to renewing biofuel cells is to replenish the anolyte suspension. It should be considered that during the operation of the BFC, the anolyte suspension inevitably changes due to the decrease in glucose concentration caused by the metabolic activity of the yeast, which can also cause the contamination of the suspension with various byproducts. In this study, to renew the BFCs, the anolyte suspensions were centrifuged after 24 h of operation to separate yeasts from PBS containing glucose and K_3_[Fe(CN)_6_], and after centrifugation, the separated yeasts were reused to prepare new suspensions needed for the construction of biofuel cells. It was found that replenishing the anolyte suspension in the BFC NiHCF/yeast or BFC PB/yeast resulted in a power density similar to the initial power density of the BFC yeast (Figure 5C). The fuel cell containing NiHCF/yeast (BFC NiHCF/yeast) showed a recovery of 77.4% of initial power density, whereas the fuel cell containing PB/yeast (BFC PB/yeast) showed a recovery of 50.1% of initial power density, indicating that NiHCF/yeast has higher viability over time than PB/yeast. These results can be interpreted as correlating with the results of yeast cell viability measurements (Figure 1), which indicate that yeast cells remain more viable after modification with NiHCF in comparison to yeast cells modified with PB. We conclude that the superior viability of NiHCF/yeast makes it a promising candidate for use in the production of regenerative yeast fuel cells. In summary, the NiHCF/yeast-based fuel cell (BFC NiHCF/yeast) and the PB/yeast-based fuel cell (BFC PB/yeast) showed that they can be renewed and remain at a satisfactory power density (>75% of the initial power density), which is an important aspect of a functioning yeast-based fuel cell.

## 3. Experimental Section

### 3.1. Materials

FeCl_3_·6H_2_O, NiCl_3_·6H_2_O, K_3_[Fe(CN)_6_], NaH_2_PO_4_, NaCl, NaOH, and methylene blue (MB) dye of ‘highest purity’ were purchased from ROTH (Karlsruhe, Germany). Platinum plates were purchased from American Elements (Los Angeles, CA, USA). Yeast cultivation YDP broth with a composition of 20 g/L dextrose, 20 g/L bacteriological peptone, and 10 g/L yeast extract was purchased from Sigma-Aldrich (Munich, Germany). Baker’s yeast (*Saccharomyces cerevisiae*) was purchased from a local bakery. Nitrogen gas (99.99%) was obtained from Elme Messer Gas (Vilnius, Lithuania). Water cleaned using the MilliQ-plius Millipore system (Milford, MA, USA) was used to prepare solutions. Phosphate-buffered solution (PBS) pH 7.1 was prepared in a 0.5 L volumetric flask by dissolving NaCl (0.1 M) and NaH_2_PO_4_ (0.01 M) into Millipore water and alkalized with NaOH up to pH value of 7.1. The accurate value of pH was determined with a HI83141 analog pH/mV/°C meter equipped with a HI1230B electrode from Hanna Instruments (Bedfordview, Republic of South Africa). The set of resistors (from 10 Ω to 94 MΩ) was bought from Lemona (Vilnius, Lithuania). The Genesys 50 UV–visible spectrophotometer was bought from Thermo Fisher Scientific (Waltham, MA, USA).

### 3.2. Yeast Cell Cultivation

The standard dry baker’s yeast was used for the experiments. Briefly, 0.5 g of dry yeast was cultivated in 20 mL of Millipore water containing 1 g of YDP broth. The flask containing yeast suspension in YDP broth was then left on a rotary shaker running at 120 rpm (rotations per minute) for 24 h at 26 ± 2 °C. After 24 h, the yeast cells were centrifuged at 1200 rpm speed for 3 min.

### 3.3. Yeast Cell Modification with Prussian Blue and Nickel Hexacyanoferrate

Briefly, 0.5 g of cultivated wet mass of yeast cells was modified with Prussian blue by adding 0.1 M of glucose, 50 mM of FeCl_3_, and 40 mM of K_3_[Fe(CN)_6_] into 5 mL of PBS pH 7.1. To modify the yeast cells with Prussian blue, the flask with yeast suspension was left on a rotary shaker for 24 h. The yeast cells’ modification with Prussian blue was completed by the separation of the yeast cells from the solution using a centrifuge at 1200 rpm speed for 3 min.

Additionally, 0.5 g of cultivated wet mass of yeast cells was modified with nickel hexacyanoferrate by adding 0.1 M of glucose, 50 mM of NiCl_3_, and 40 mM of K_3_[Fe(CN)_6_] into 5 mL of PBS pH 7.1. To modify the yeast cells with nickel hexacyanoferrate, the flask containing the yeast suspension was placed on a rotary shaker for 24 h. The yeast cells’ modification with nickel hexacyanoferrate was completed by the separation of the yeast cells from the solution using a centrifuge at 1200 rpm speed for 3 min.

### 3.4. Yeast Cells’ Viability Measurements

The spectrophotometric method [26] described by A. Matsumoto et al. was used to determine the viability of unmodified yeast, NiHCF/yeast, and PB/yeast. A solution containing 0.03 mM MB and 68 mM trisodium citrate in water was used. Briefly, 0.05 g of freshly grown unmodified yeast, NiHCF/yeast, or PB/yeast was mixed with 100 µL of MB solution by vortexing. The yeast cells were immediately separated from the solution by using a centrifuge (1200 rpm for 3 min), and 10 µL of the residual solvent was diluted with 1 mL Millipore water. The optical spectra of the supernatant were plotted, and the absorption values were registered at the interval of wavelengths from 550 nm to 750 nm. The absorption peak value at 665 nm was used for the calculation of yeast viability [26]. For control experiments, non-viable yeast, unmodified yeast, NiHCF/yeast, and PB/yeast were kept at 100 °C in an oven for 30 min before being suspended in a solution, hereafter referred to as “base solution”, containing methylene blue and trisodium citrate. Spectrophotometric measurements were carried out by using Thermo Fisher Scientific spectrophotometer Genesys 50 (Waltham, MA, USA).

### 3.5. Electrochemical Measurements

Electrochemical measurements of linear sweep voltammetry were performed by using µAUTOLAB potentiostat/galvanostat from ECO-Chemie (Utrecht, The Netherlands). A three-electrode system was used in which a Pt plate with a geometric area of 1 cm^2^ was used as the working electrode, a Pt plate with a geometric area of 1 cm^2^ was used as the counter electrode, and the saturated silver–silver chloride (Ag|AgCl|KCl_sat_) electrode was used as the reference electrode. Prior to all electrochemical measurements, the working and counter electrodes were prepared for measurements by cleaning the electrodes with Vienna lime and rinsing them with Millipore water.

Linear sweep measurements were carried out at room temperature and atmospheric pressure. During these measurements, the working, counter, and reference electrodes were immersed in the electrochemical cell filled with 10 mL of suspension. A suspension (10 mL) was prepared by adding 5 mM K_3_[Fe(CN)_6_], 30 mM glucose solution, and 0.1 g/mL yeast (unmodified, NiHCF/yeast, or PB/yeast) to PBS pH 7.1. Before and between measurements, an electrochemical cell containing the suspension was deoxygenated by flushing with inert N_2_ gas for 30 min. All linear sweep measurements were performed at a scan rate of 50 mV/s in the potential range from the equilibrium potential of −0.3 V to the potential value of 0.8 V (vs. Ag|AgCl|KCl_sat_).

### 3.6. Design and Investigation of the Biofuel Cell

For the design of the biofuel cells (BFCs), two Pt plate-like electrodes with a geometric area of 1 cm^2^ were used, each of which was immersed in solutions of different compositions. The Pt electrode immersed in 10 mL of PBS pH 7.1 containing 5 mM of the charge transfer mediator K_3_[Fe(CN)_6_], 30 mM of glucose, and 0.1 g/mL of unmodified yeast, NiHCF/yeast, or PB/yeast was used as the anode. The Pt electrode immersed in 10 mL Milli-Q water containing 5 mM of electron acceptor FeCl_3_ was used as a cathode. The volume of the catholyte and anolyte was 10 mL. In this process, FeCl_3_ accepts the electron and is reduced to a FeCl_2_ compound.

A charge transfer mediator, [Fe(CN)_6_]^3−^, was selected for use in the anodic compartment of the BFCs because [Fe(CN)_6_]^3−^ ions are capable of accepting electrons produced by the yeast–glucose metabolic reaction, i.e., performing the metabolic function of oxygen [27]. However, [Fe(CN)_6_]^3−^ has a lower standard reduction potential than oxygen; thus, [Fe(CN)_6_]^3−^ can transfer an electron to the anode more easily than oxygen, improving the efficiency of the yeast-based BFCs. Therefore, to prevent electron transfer from yeast enzymes to oxygen in the solution, the anodic side of the BFCs (containing yeast cells) was under the influence of inert gas (N_2_) during the experiment.

**Figure 6:**
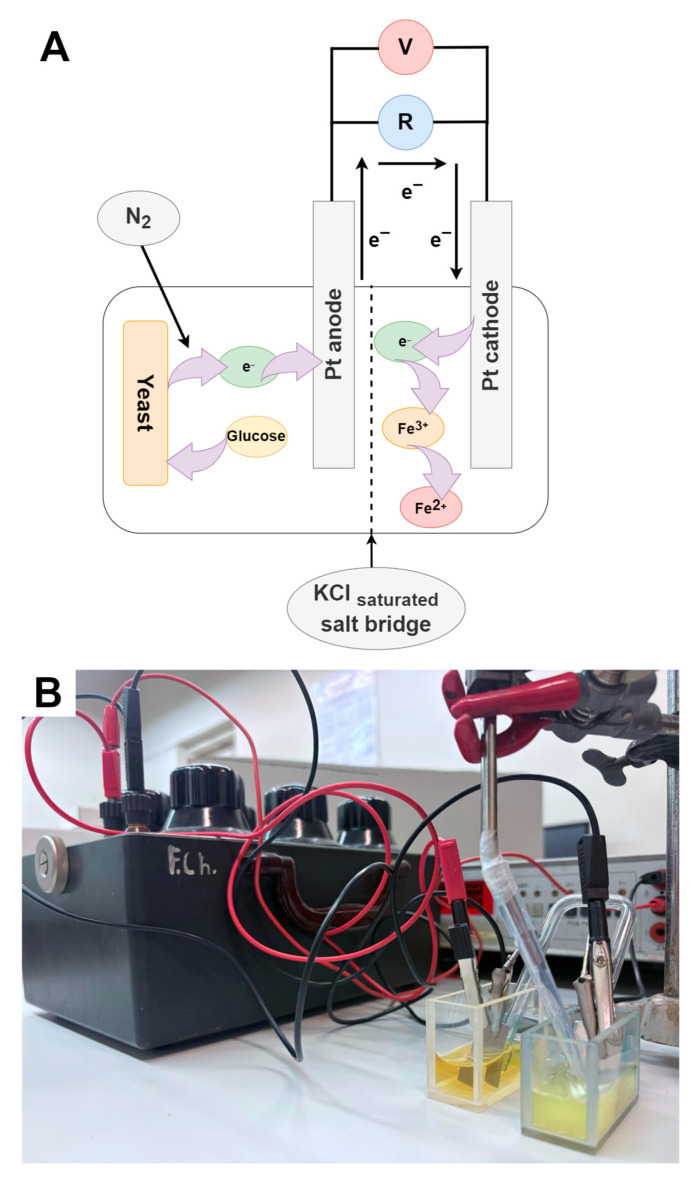
(**A**) Schematic representation of the construction of the biofuel cell based on *Saccharomyces cerevisiae* cells; (**B**) the picture of the BFC constructed according to scheme A.

Both sides of the BFCs were connected by using a salt bridge containing a saturated KCl solution. A range of resistors from 10 Ω to 94 MΩ were placed between the cathodic and anodic sides of the BFC. The generated voltage was measured by using a “Sigma 33A TRMS” multimeter (Pittsburgh, PA, USA). Figure 6 shows a schematic representation (Figure 6A) and picture (Figure 6B) of the BFCs studied.

## 4. Conclusions

A simple and inexpensive method of chemically modifying baker’s yeast with nickel hexacyanoferrate (NiHCF) and Prussian blue (PB) is presented in this study. The modification of yeast cells with NiHCF was found to be less detrimental to cell viability than modification with PB, as approximately 100% of NiHCF/yeast cells were viable compared to 61% of PB/yeast cells.

Voltammetric analysis showed that both the NiHCF/yeast and PB/yeast could achieve a higher anodic current of Fe(CN)_6_]^3−^ oxidation than unmodified yeast cells. The constructed biofuel cells based on the NiHCF/yeast and PB/yeast cells resulted in higher power densities (62.66 mW/m^2^ and 94.09 mW/m^2^, respectively) than those obtained with unmodified yeast cells containing biofuel cells (42.25 mW/m^2^). The proposed method for the modification of the yeast cell, which resulted in an increase in the electrical power of the yeast fuel cell, is in line with other studies [17,30,31,32]; however, in this study, we found that PB/yeast and NiHCF/yeast can be used for prolonged operation (at least 24 h) of a biofuel cell, as refreshing the glucose-containing PBS solution of the yeast suspension resulted in maintaining 77.4% (NiHCF-modified yeast) and 50.1% (PB-modified yeast) of the initial power density.

The present study demonstrates that yeast modification with PB markedly enhances the power generated by yeast-based biofuel cells, with an approximately 2.2-fold increase (compared to unmodified yeast cells). Furthermore, yeast modification with NiHCF represents an effective strategy to mitigate the deleterious effects of chemical modification on yeast cell viability and the longevity of yeast-based fuel cells, as modification of yeast cells with NiHCF did not affect yeast cell viability. Our future goal is to investigate the possibility of immobilizing the PB- and NiCHF-modified yeast on electrodes using fixation in redox polymers and to apply these electrodes in the production of electricity from food industry wastewater.

## Data Availability

The data presented in this study are available on request from the corresponding author.
